# Exploring How the Psychological Safety of Patients Is Impacted by Restrictive Practices in Inpatient Mental Healthcare: A Qualitative Study

**DOI:** 10.1111/inm.70148

**Published:** 2025-10-29

**Authors:** Bethany Griffin, Judith Johnson, Katharina Sophie Vogt, Emily Mizen, Chris Keyworth, John Baker

**Affiliations:** ^1^ School of Psychology University of Leeds Leeds UK; ^2^ Bradford Institute for Health Research Bradford Royal Infirmary Bradford UK; ^3^ Division of Nursing, Midwifery and Social Work, Faculty of Biology, Medicine and Health, School of Health Sciences University of Manchester Manchester UK; ^4^ Manchester University NHS Foundation Trust Manchester UK; ^5^ School of Public Health and Community Medicine University of New South Wales Sydney Australia; ^6^ Department of Psychology, Institute of Population Health University of Liverpool Liverpool UK; ^7^ Norwich Medical School University of East Anglia Norwich UK; ^8^ School of Healthcare University of Leeds Leeds UK

**Keywords:** coercion, inpatient, mental health, patient safety, restrictive practice

## Abstract

Restrictive practices are used to contain risk and maintain physical safety on inpatient mental health wards, but have been shown to negatively impact patient well‐being and trust. Researchers and professionals have suggested that inpatient mental healthcare focuses on physical safety at the expense of psychological safety. The relationship between restrictive practices and psychological safety has not yet been explored. This study aimed to explore the impacts of receiving and witnessing restrictive practices on psychological safety, to understand what could be done to make restrictive practices psychologically safe. Eighteen semi‐structured interviews were carried out with former patients (aged 20–60 years) who had been discharged for longer than 6 months from adult inpatient mental healthcare in the United Kingdom. Data were analysed using reflexive thematic analysis. Four themes were generated: (1) Reactive over proactive care: seeing the behaviour and not exploring the reason, (2) A chaotic environment cannot provide safety for patients and staff, (3) Psychological impact of the (perceived) power imbalance between staff and patients and (4) Emotionally all in it together, for better or worse. The results support that physical risk is heightened in inpatient settings, but containing this should not come at the expense of psychological safety. Supportive communication and giving small acts of control to patients should be prioritised to enhance the psychological safety of patients.

## 1 Introduction

Patient safety, the absence of avoidable harm and reduction of unnecessary risk to patients, is a priority for healthcare organisations worldwide (World Health Organization 2023). Safety incidents encompass falls, medication errors and adverse drug events (Cuomo et al. 2020). On mental health wards, additional considerations to protect the physical safety of people on the ward are needed because of complexities related to patient presentations, including potential physical and mental health multi‐morbidities and risk of aggression (D'Lima et al. 2017). Restrictive practices and adaptations to the environment are therefore used for the physical safety of patients and staff. After consultation with a lived experience advisory group, the term patient will be used throughout this article to refer to people receiving inpatient care.

Many inpatient mental health wards in the United Kingdom (UK) are rated as ‘required improvement’ or ‘inadequate’ for safety, including 77% of NHS Trusts and 59% of independent sector organisations caring for adults on acute wards and PICUs (Department of Health and Social Care 2024). Unsafe wards have an overreliance on restrictive practices to contain risk, demonstrating an overwhelmed and stretched environment (Care Quality Commission [CQC] 2024). Restrictive practices are deliberate acts that restrict movement, liberty, and/or freedom to take control of a potentially dangerous or harmful situation (Department of Health 2014; National Institute for Health and Care Excellence [NICE] 2015); also referred to as coercion and restrictive interventions. Practices include intrusive methods such as restraint, seclusion, observations and rapid tranquilisation, and practices applied throughout a ward, like locked doors and blanket restrictions (i.e., limitations on belongings and ward rules).

The use of restrictive practices is controversial, particularly the use of physical measures. Both patients and staff have reported physical and psychological harm from restrictive practices (Butterworth et al. 2022). Patients report feeling controlled, traumatised and fearful when restrictive practices are used (Bendall et al. 2022; Scholes et al. 2022). As a result, there have been interventions developed with the aim of reducing the use of and need for restrictive practices (i.e., Safewards and Six Core Strategies; Bowers et al. 2015; Huckshorn 2004). Staff acknowledge the distress that restrictive practices can cause but have expressed concerns for complete abolition and the reliance on practices in high‐risk situations (Gerace and Muir‐Cochrane 2019; Snipe and Searby 2023). There appears to be a disconnect between keeping patients physically safe whilst protecting them from psychological harm in inpatient care.

## 2 Background

Safety in mental health care is often defined physically, focusing on quantifying and reducing risk and incidents (Delaney and Johnson 2008; Thibaut et al. 2019). Managing risk is necessary in high‐stress situations (i.e., posing a risk to life) but can lead to psychological harm when incidents are generalised to patient behaviour (Bendall et al. 2022; Tully et al. 2022). Safety for both patients and former patients means a combination of physical and psychological safety (Berzins et al. 2020). Lack of psychological safety in Berzins et al. (2020) referred to experiences that led to fear, distress and/or psychological harm. Qualitative research has identified factors that impact the psychological safety of patients in inpatient mental health settings (Cutler et al. 2021; Vogt et al. 2024). Enhancing factors included: being physically safe, positive relationships with staff, patient choice and having access to meaningful occupation. Detrimental factors included: use of methods to contain physical safety, negative relationships with staff, and not being involved in decisions around care. Patient‐focused research in this area is currently in its infancy. Therefore, identified factors of psychological safety are broad and require further exploration.

Discrepancies between organisational priorities, patient needs and staff capabilities need to be addressed. Patient safety priorities do not consider that patient safety means psychological safety to patients (Berzins et al. 2020). How this looks in practice should be explored further. The reduction of restrictive practices is seen as improving patient safety, and Bowers et al. (2015) demonstrated a link between employing alternative psychologically informed approaches and reducing restrictive practices. However, in practice, staff do not consistently have psychologically safe alternatives available to do this, especially in crisis situations. How restrictive practices are used in crisis situations needs to be explored to mitigate long‐lasting psychological impacts. Research exploring the patient experience of restrictive practices is growing; however, there is limited research on the impact of witnessing restrictive practices from the perspective of patients (Wilson et al. 2017). Understanding the experience of restrictive practices first‐hand and witnessing incidents with peers is important for overall safety on inpatient mental health wards.

No research to date has explored the relationship between restrictive practices and psychological safety explicitly. It is imperative that the lived experience voice be considered when setting priorities for patient safety research. Therefore, this study utilised an exploratory qualitative approach, interviewing former mental health inpatients (discharged for longer than 6 months) in the UK. This study aimed to explore the impacts of receiving and witnessing restrictive practices on psychological safety to understand what could be done to make restrictive practices psychologically safe.

## 3 Method

### 3.1 Design

An exploratory qualitative study, which used semi‐structured interviews, was conducted.

### 3.2 Sampling and Recruitment

Adults with experience of restrictive practices in UK inpatient mental health services (discharged for longer than 6 months) were eligible for participation. Former patients of forensic units and whose only experience was in child and adolescent services, were not eligible to participate. Participants had to be able to provide informed consent. This study utilised volunteer sampling where potential participants volunteered to participate (Gill 2020).

The study was advertised on the social media platform X, using a study poster. The study poster contained information about the study and listed restrictive practices as: segregation, locked doors, seclusion, restraint, rapid tranquilisation, coercion and compulsion related to treatment and observations. The recruitment method allowed participants who were no longer in touch with mental health services to take part. During recruitment, participation from people who were male and/or from ethnic minority backgrounds was purposefully requested.

### 3.3 Ethics Statement

Ethical approval was granted by the University of Leeds, School of Psychology Ethics Committee (20/07/2023; reference PSCETHS‐674). Consent for this study was obtained using Qualtrics to reduce the burden on participants of printing and returning the consent form. After the interview, participants were sent a debrief email containing a £30 voucher code for their participation.

### 3.4 Procedure

Interested participants emailed the lead researcher, and eligible participants were provided with an information leaflet. Semi‐structured interviews were carried out online through MS Teams and were recorded for transcription purposes. A topic guide was established through reviewing the literature and the research team expertise (mental health nursing, clinical psychology and lived experience). People with lived experience were involved in the development of research materials, topic guide development and conceptualisation of psychological safety. This was achieved through the inclusion of lived experience researchers on the research team and a review of the findings by an independent lived experience advisory group. The full topic guide is presented in the supporting material (Appendix S1).

Participants were encouraged to discuss any ward experience that they deemed restrictive. KV conducted the first three interviews, due to previous experience with psychological safety research, while BG observed. BG then carried out the remaining 15 alone. The decision to end recruitment was made based on a pragmatic decision that a breadth of restrictive practices and experiences had been discussed. Interviews were transcribed verbatim using MS Teams and then checked/edited by the lead researcher to ensure accuracy.

### 3.5 Analysis

Braun and Clarke's Reflexive Thematic Analysis (RTA; Braun and Clarke 2021a) guided the analysis, providing a contextual and situational reflection of former patients' experiences (Braun and Clarke 2021b). The six phases of analysis using Braun and Clarke's RTA (2021a) were followed. A critical realist approach to analysis was taken, using a combination of latent and semantic coding.

The first author (BG), a female PhD student with no prior experience working in or being a patient of mental health services, led the analysis. This work was supervised by a mental health nurse (JB) and a clinical psychologist (JJ), both of whom have extensive research experience. They both contributed to the generation of themes and theme development. The full process is detailed in the supporting material (Appendix S2).

The rest of the research team comprised two trainee clinical psychologists (KV and EM) and a chartered psychologist (CK), all of whom have experience conducting qualitative research. Members of the research team had lived experience, but to protect the researcher confidentiality, no identifying initials are provided.

## 4 Results

Thirty‐four former patients expressed interest through email, with 20 meeting eligibility criteria and providing consent. Two people did not attend their interview, meaning there were 18 participants, with a mean age of 38.1 years (range: 20–60 years). The participants were predominantly female (*n* = 13). Fifteen participants were White‐British, one participant was White‐Irish, one participant was from a Roma background, and one participant was from a mixed ethnic background. Participants had experience in inpatient care in a range of areas across the UK. Eleven participants were employed full‐time, nine of whom worked in mental health services, healthcare or social work. None of the participants had received a post‐incident debrief following restrictive practices that they deemed adequate. Only one participant had received a debrief after witnessing restrictive practices on the ward. See Table 1 for additional information about the participants' experiences of restrictive practices. Interviews ranged from 23 to 70 min and averaged 51 min. The total data collected amounted to 15 h, 21 min.

**TABLE 1:** Participant overview and quote identification.

Participant ID	Gender	Age range	Occupation	Most recent inpatient stay	Restrictive practices discussed	
		(Years)		(Year)	Experienced	Witnessed
Participant 1	Female	26–35	Doctor	2015	1:1 Observations seclusion rapid tranquilisation Sectioned mid‐stay	Restraint
Participant 2	Female	36–45	Student adviser	2021	1:1 Observations Restraint	Restraint
Participant 3	Female	46–55	Unemployed	2006	Restraint Rapid tranquilisation	Coercive language Locked doors
Participant 4	Female	26–35	Postgraduate student	2018	1:1 Observations Locked doors	Restraint Restraint for nasogastric (NG) feeds
Participant 5	Female	46–55	Senior caseworker	2021	Blanket restrictions Coercive language Locked doors Segregation	1:1 Observations
Participant 6	Non‐binary	36–45	Unemployed	2018	Restraint Seclusion Sectioned mid‐stay	Restraint
Participant 7	Female	36–45	Administrator and expert by experience	2020	Rapid tranquilisation Coercive language	Restraint
Participant 8	Female	18–25	Unemployed	2021	Restraint rapid tranquilisation 1:1 Observations blanket restrictions	Restraint
Participant 9	Male	46–55	Researcher	2019	Locked doors coercive language	Restraint rapid tranquilisation
Participant 10	Female	18–25	Doctor	2022	Restraint rapid tranquilisation removal of belongings locked doors	Restraint
Participant 11	Male	55–65	Team manager	2013	Rapid tranquilisation observations	N/D
Participant 12	Female	36–45	Emergency care assistant	2021	Restraint rapid tranquilisation seclusion	Physical handling
Participant 13	Female	18–25	Healthcare	2023	2:1 Observations ‘Treated under restrictive practice’ restraint CCTV observations	Restraint
Participant 14	Female	46–55	Social worker	2022	Seclusion coercive language	Segregation locked doors
Participant 15	Female	46–55	Full‐time carer	2002	Seclusion rapid tranquilisation	Seclusion
Participant 16	Non‐binary	26–35	Student and project co‐ordinator	2017	Observations locked doors blanket restrictions	N/D
Participant 17	Non‐binary	36–45	Unemployed	2021	Rapid tranquilisation blanket restrictions	Restraint
Participant 18	Female	18–25	Student	2022	1:1 Observations restraint	Restraint

### 4.1 Reflexive Thematic Analysis

Four themes were generated:

1. Reactive over proactive care: seeing the behaviour and not exploring the reason for it
2. A chaotic environment cannot provide safety for patients and staff
3. Psychological impact of the (perceived) power imbalance between staff and patients
4. Emotionally all in it together, for better or worse

Quotations are included to provide evidence for the findings in participants' own words.

#### 4.1.1 Theme 1. Reactive Over Proactive Care: Seeing the Behaviour and Not Exploring the Reason for It

Restraint and observations were deemed necessary to keep patients alive in high‐risk situations (self‐harm incidents and suicide attempts), but participants felt that staff often used them without insight into the cause of the behaviour. The detachment of ‘observed’ behaviour from underlying feelings and thoughts contributed to participants feeling less psychologically safe. Pacing or expressing frustration often resulted in sanctions (e.g., discussions at ward round, cancellation of leave and restrictions of activity involvement) without discussion with staff to understand the cause. This resulted in participants feeling responded to with violence and made the ward an unsafe space for honest expression of thoughts and feelings.

‘I had made attempts to harm myself and that's about coping with feelings of distress and assuming that's now recognised…but that just escalated things even further’. **Participant 2**

In addition, locked wards made participants feel trapped in an unsafe space. For many, this impacted their progress as they would often ‘shut down’ to avoid interactions with staff. Combined, distress that led to the initial use of restrictive practices was perpetuated.

‘I guess, what staff are trying to do is to keep everyone safe. But then, if you're in that situation, the patient, the fact the doors are locked makes you feel unsafe because you're locked in this situation’. **Participant 14**

Participants interpreted physical intervention (seclusion, restraint and/or rapid tranquilisation) as a negative reaction to their emotions. The restrictions were viewed as disproportionate and immediate, with little consideration for prior de‐escalation. The experience could be described as reactive care, interventions used quickly for convenience, rather than proactive, considering the context and de‐escalation.

‘I do think there is a kind of default to restrictive practice actually. And yeah, there's all this sort of rhetoric around is the step of last resort and I don't think that's true in practice’. **Participant 9**

For many participants, psychosis was experienced as fear, danger and related to the belief that others were trying to harm them. When psychosis was not considered as cause for a patient's ‘risky behaviour or discussed openly, staff became ‘real‐world’ manifestations of psychotic experiences. Here, restrictive practice use was interpreted as an intention to cause harm and injury, making it difficult for participants to feel physically and psychologically safe.

‘Having and feeling like someone was gonna be grabbing me, to then having actual people that I believe were supposed to be there to help me, then almost turning against me and like treating me like I was the problem. Yeah, I felt really unsafe’. **Participant 12**

Reactive restrictive practices left participants alone with negative emotions and impacted psychological safety. Vulnerable and frightening experiences with staff had repercussions for the therapeutic alliance and participants' emotional well‐being. When restrictive practices were about to be used, communication was prioritised in some instances, which supported psychological safety and relationships between patients and staff.

‘She [staff] was able to kind of make that assessment and seeing it was just a bit over the top and that it didn't need to be done. I feel so much better for the fact that she'd actually like listened and could see that me having my shower was actually gonna help me’. **Participant 4**

#### 4.1.2 Theme 2. A Chaotic Environment Cannot Provide Safety for Patients and Staff

Participants described an unrelenting chaos on the ward, with little opportunity for refuge to decompress amongst the waves of incidents. Exclusion from decisions, particularly after restrictive practices had been used, caused upset and further incidents. Restrictive practices were frequently made more psychologically harmful by compounded layers of unwritten rules and inconsistencies in decisions and skills of practitioners. An environment and culture based on uncertainty and chaos limited participants' capacity to feel psychologically safe.

‘… it's that thing of feeling on edge all the time and not being able to … just be able to get on with things.’ **Participant 1**

Disagreements between staff (i.e., nurse and doctors, and between nurses), created a culture of uncertainty and led to questions about staff competence and decision‐making ability. Lack of faith in care providers, meant the safety of participants and peers was questioned. The working environment was described as unpredictable and stretched. Examples given included: not having enough staff on shift and no time to communicate effectively with patients.

‘It's really chaotic. It didn't make me feel unsafe for me. It made me feel unsafe for other people, like for months I was saying someone is gonna get seriously hurt or killed… it's very understaffed, it's stretched to capacity … as an incident is going on, you can't just leave like you're locked in there with it and there's alarms going off and you worry for that person… It didn't make me feel unsafe, but I just recognised that my home was unsafe, you know?’. **Participant 18**

Participants felt compassion for staff and mentioned that working in a psychologically safe way cannot be prioritised in that environment. The chaos translated into an unpredictable and custodial dynamic between staff and patients. Without a strong therapeutic relationship and safe environment, treatment decisions came across as ill‐informed, not considered and resulted in a default to restrictions.

‘If you generate a culture that is impersonal, a ‘doing to’ culture, it's not a particularly empathic culture. You are gonna end up doing stuff to people without questioning it…I think in that environment, that's not gonna be conducive to recovery, right?’ **(Participant 11)**

#### 4.1.3 Theme 3. Psychological Impact of the (Perceived) Power Imbalance Between Staff and Patients

Restrictive practices were seen as reflections of staff's frustrations towards participants and were often viewed as a medium for staff to demonstrate their power. Staff had the power to remove belongings and strip rooms, which was interpreted as ‘callous and cruel’ *(Participant 6)*. Participants then felt punished, ‘bad’ and guilty for behaviour that was caused by their symptoms and distress. Participants often felt that staff underestimated the weight of their actions.

‘I was there because I was unwell, not because I was bad… I think in hospital you're just made to feel that you're bad’. **Participant 2**

Meaningful occupation and belongings were seen as game pieces in the power play between staff and patients, particularly when the decisions for permitting or withholding them were not explained. To regain power, participants would rebel and act in atypical ways.

‘And because staff were, like, making what felt like really arbitrary decisions without actually telling me any explanation or why they thought it was important, I suddenly wanted to do all the things I couldn't do’. **Participant 16**

Belongings brought from home were among the few things that participants had a degree of control over, but in some hospitals, they were removed. When these were removed, so was their safety.

‘I think if you're not giving people choice and things, sort of try to give them small elements of control’. **Participant 1**

Blanket restrictions around personal items, that is, razors, items of clothing that had drawstrings or electrical cables on the wards, were used irrespective of individual risk. This was interpreted as another way for staff to control patients. Safe items being labelled as unsafe items often increased the perception of personal risk when participants received their items back at discharge. To participants, this felt counter‐intuitive and changed the view of safety for the items once discharged.

‘…to then be discharged to the community a couple of months later, having had no access to any of my belongings, the entire time. And then a few months later, I've been discharged with everything in my suitcase’. **Participant 8**

Containment methods to keep patients physically safe in mental health settings would not be acceptable in any other context. Participants often viewed their circumstances in contrast with someone that had not been an inpatient.

‘If somebody in the community was like locked in a room and been made to take medication, that would be really traumatic for them. So it isn't any different if you're in hospital and have a mental illness. It's still and if anything, probably it's more traumatic because you're already sort of scared…hearing voices on top of it would be scary for anybody’. **Participant 14**

Dignity of care and control was compared to receiving treatment in a physical healthcare setting. Being on observations and using the bathroom or not having access to a toilet in a seclusion room was a traumatising and upsetting experience for many participants. Participants felt that staff were not aware of the impact that lack of dignity and control over personal decisions had on psychological safety.

‘So it was just mimicking the fear that I already had and comparing that. Like the fact that I couldn't use a toilet and that I had all my clothes taken off me, I wasn't even asked if I would undress. They just ripped them off me and it was it just. Yeah, it just I felt so unsafe’. **Participant 12**

#### 4.1.4 Theme 4. Emotionally all in It Together, for Better or Worse

Relationships on the ward added complexity to the inpatient experience; they were intensified but also had the potential to make participants feel psychologically safe. Constant proximity to staff and other patients complicated the development of positive relationships. The weight and importance of relationships on the ward felt increased and led to participants being more invested in the lives of others. Having friends or peers to relate to allowed for shared experiences and peer support. The increased importance of relationships meant that upsetting peer‐staff interactions impacted participants' own psychological safety and caused fear for the physical safety of their peers. Upset and distress when their peers were restrained or separated from the ward was common.

‘You might be close to and support each other, and then yeah, to have to see that [restrictive practices] is…that can be difficult at times and that was certainly something that‐ I wouldn't sleep. I'd be in tears for days’. **Participant 8**

Peer relationships were frowned upon by staff, with participants being told they were too involved in their peer's care. An ‘us vs them’ mindset was common, where participants supported and defended peers against staff, often resulting in further *conflict*.

‘No, no, because if I even tried to raise it, it was we can't talk about the patients and so you couldn't, and it was it was me versus them’. **Participant 3**

Close relationships with staff members were beneficial for most participants. However, when staff behaviour did not meet participants' expectations, it resulted in upset and setbacks to the therapeutic relationship. Staff who worked to promote the psychological safety of patients through empathetic and individualised care did not ‘fit in’ with the ward's culture and often left. As a result, participants felt hopeless and psychologically unsafe when they thought about the future of their care, especially when they had been subject to multiple restrictions.

‘You've got good staff members who are nice, it's really hard for them in a culture where everybody else is in on it [ward culture] to try and make a difference or to stand up’. **Participant 16** .

Communication with staff was often limited, meaning psychological safety and trust were low before restrictive practices were used. This also meant that communicating decisions in the ‘wrong way’, that is, through posters or in ward rounds, came across as threatening. Communication, when done in a positive and individualised way, could strengthen the therapeutic relationship and psychological safety before and after restrictions.

‘…possibly if I'd been given some time to talk with somebody, I wouldn't have become sort of so agitated and distressed. I don't know, it wouldn't have changed the situation, but maybe I would have felt that I was being listened to’. **Participant 14**

The wards were described as closed environments that only people who have been in, could understand. Communication with family and peers was hard after discharge, as they had not experienced inpatient treatment. Not having a post‐incident debrief made leaving the ward harder. Several participants were discharged when they were still on observation or had stripped rooms.

‘It made it much harder when I left the ward environment to suddenly have freedom. I think it made it a bigger deal. Not being able to build up my own level of trust in my own ability to keep myself safe. So when I then went out, I was I was far riskier’. **Participant 5**

### 4.2 Psychological Safety

Based on this thematic work, psychological safety was conceptualised as, ‘Feeling validated in your experience of the world and the belief you will be treated fairly based on your individual needs. Being psychologically safe provides protection from lasting psychological harm from your environment. It's not just about being physically safe but being protected from events that may have lasting effects in the future’. The development process and lived experience collaborator feedback are outlined in the supporting material (Appendix S3).

## 5 Discussion

This is the first qualitative study to explicitly explore the relationship between restrictive practices and psychological safety in UK inpatient mental health settings. There are four key findings. First, perceived risk is heightened on mental health wards due to concerns over patient aggression, self‐harm and suicide attempts. Containing risk should not come at the expense of the psychological safety of patients. Psychological safety and physical safety are interlinked; patients cannot have one without the other. Second, restraint and observations were seen as necessary in crisis situations, but can easily lead to fear, distress and compounded trauma when psychological safety is not considered. Third, the physical safety of patients was seen to be emphasised throughout the inpatient process, ignoring psychological safety, individual recovery progress and the participant's perception of their risk. Fourth, communication and empathetic care have the potential to foster psychological safety when restrictive practices are used.

The current work extends the literature on the experience of restrictive practices. Psychological safety is not just impacted by physical measures but also by blanket restrictions (locked doors and ward rules). Research in this area predominantly focuses on the impacts of formal measures (Askew et al. 2019; Butterworth et al. 2022; Larue et al. 2013), meaning measures such as blanket restrictions and ward rules are considered less harmful in practice. The present study extends previous knowledge by demonstrating that psychological harm to patients is not limited to restraint, seclusion and rapid tranquilisation.

Perceived power imbalances have negative impacts on the psychological safety of patients and can create a vicious cycle of incidents. Inpatient mental health settings are naturally coercive environments because of the Mental Health Act (Department of Health 2015). Locked doors, restraint and ward rules lead to patients feeling disempowered and not trusted (Cusack et al. 2018; Missouridou et al. 2022; Pelto‐Piri et al. 2019). The current study demonstrates that an environment fuelled by containment and misuse of power leads to miscommunications and a breakdown in trust. It is important to consider how the lack of trust and resulting behaviours impact the recovery of patients and relatio6nships with care providers.

Uncertainty around when restrictive practices were going to be used impacted mood and caused ‘risky’ behaviours (described by participants as pacing the wards and appearing anxious). Bowers (2014) suggested the idea of containment (restrictive practices) and conflict (patient and staff negative interactions) as having a dynamic reciprocal relationship, whereby containment can give rise to conflict instead of defusing it. In the current study, the uncertainty about when restrictive practices would be used resulted in further conflict. There is scope to expand and develop existing interventions by explicitly considering the use of restrictive practices (i.e., ward rules) as a ‘flashpoint’ for further restrictions.

Positive therapeutic relationships are interpreted as psychologically safe care. A positive relationship with care providers, built on good communication, is known to have a strong impact on the outcomes of patients in mental healthcare, such as wellbeing, recovery and engagement with services (Hartley et al. 2020; Mcandrew et al. 2014; Moran et al. 2014). Communication techniques (i.e., de‐escalation and debriefing) have been developed (Celofiga et al. 2022; Grundy et al. 2024; Price et al. 2024) and are recommended in relevant UK policy guidance when restrictive practices are used (NICE Guideline 10; NICE 2015 and NICE Quality Standard 154; NICE 2017). However, the current work and previous research suggest that de‐escalation methods are not always utilised in practice, suggesting there is a need to consider how relevant policies can be translated into usable guidance (Inglis and Clifton 2013; Sustere and Tarpey 2019).

### 5.1 Implications for Practice

Organisations should support training and use of evidence‐based resources to move away from reliance on restrictions and containment to psychologically safe care. Priority should be on training around patient risk and appropriate responses to behaviour. Similarly, systemic issues that allow unsafe practices to happen should be addressed. Organisations should work to promote the psychological safety of staff to ensure they feel comfortable speaking up about unsafe practices (Hunt et al. 2021; O'Donovan et al. 2021).

Staff should be aware of the lasting impacts care practices can have on patients. All participants of the current study described the lasting impacts restrictive practices had on their lives post‐discharge. None of the participants in the current study received an appropriate post‐incident debrief, which has likely caused lasting harm. Iatrogenic harm means harm resulting from medical care and typically refers to physical harm caused by treatment (e.g., adverse drug reactions; Sampath 2022). Iatrogenic harm can mean psychological harm resulting from interactions and restrictions placed on patients for their physical safety (Downs 2024).

Restrictive practices can be viewed as an abuse of staff power. It is acknowledged that there are legal implications in the use of restrictive practices through the Mental Health Act (Department of Health 2015). Giving small acts of control to patients could improve perspectives on staff and restrictive practices. In practice, this could be achieved through allowing choice over small decisions, discussing preferences for de‐escalation techniques, considering and actioning patient feedback, and allowing open and honest communication around ward rules through community meetings.

### 5.2 Implications for Future Research

As participants recognised the need for restrictive practices in some cases to maintain their physical safety, reducing restrictive practices without providing psychologically safe care (care where the patient feels supported and validated in their experience) could mean that patients feel at risk to themselves. Further exploration of what providing psychologically safe care looks like when restrictive practices must be used for the physical safety of patients could mean that harm from these practices is minimised.

Further research into relationships on the wards is needed. In inpatient mental health, it is important that the confidentiality of patients be respected, but the closed nature of the ward causes overlap in patient experience. The current work has demonstrated the complexity of relationships and the impact they have on the psychological safety of patients. Further exploration of how patients and staff could be supported to build healthy and respectful relationships with one another is needed. This could potentially reduce the number of incidents and the need for restrictive practices.

### 5.3 Limitations

There are four main considerations to note. First, participants were recruited using volunteer sampling. It is possible that the sampling method resulted in sampling bias, meaning participants who were more likely to have had negative experiences. Second, the study recruited previous patients who had been discharged for longer than 6 months. As there were no limitations on how long they had been discharged, it is possible that there could have been issues with participant recall. Third, while the current study did address the first‐hand experience of restrictive practices, there was limited discussion of the witnessed restrictions in comparison. While the researchers expected the discussion to be limited to explicit restrictions, participants mainly discussed their experience of witnessing the restraint of other patients. Fourth, half of the participants had experience working in health and social care roles. This could have impacted their patient experience and reflections after discharge; thus, it should be considered when interpreting the results.

## 6 Conclusions

This thematic account of former patients provides an insight into how restrictive practices can leave patients feeling like ‘risks’ rather than people and that psychological wellbeing is often ignored in inpatient settings. A move towards the rehabilitation of patients seeking care for their mental health is needed. Supporting people with their symptoms and experiences, whilst helping them to feel safe, should be a priority over containment and restrictions.

### 6.1 Relevance for Clinical Practice

Ensuring that patients are physically and psychologically safe whilst receiving mental health care is important. This study demonstrates the impact that restrictive practices have on psychological safety, from the perspective of former patients and starts to identify key areas for improvement. Improving the ways restrictive practices are conducted is crucial to enhancing psychological safety in patients. Improvements could include giving small acts of control to patients, increasing consistency and certainty around the use of practices, creating calm ward environments, enhancing communication between patients and staff, and promoting positive relationships on the ward.

## Author Contributions

The authors confirm their contribution to the paper as follows. Conceptualisation and design: B.G., J.B., J.J. and K.S.V.; development of research materials: B.G., E.M., J.B., J.J. and K.S.V.; data collection: B.G. and K.S.V.; analysis: B.G. led analysis and J.B. and J.J. subsequently contributed; draft manuscript preparation: led by B.G. and J.B., J.J. and C.K. subsequently contributed. All authors had the opportunity to review the results and approve the final version of the manuscript.

## Ethics Statement

Favourable ethical approval was granted by the University of Leeds, School of Psychology Ethics Committee (20/07/2023; reference PSCETHS‐674).

## Consent

Participants understood that the experiences that they shared in the interviews would be included in the paper and gave consent for anonymised/unidentifiable quotes to be used.

## Conflicts of Interest

The authors declare no conflicts of interest.

## Supporting information


**Appendix S1:** Topic guide.
**Appendix S2:** Analysis considerations and procedure.
**Appendix S3:** Iterations and development process of the definition of psychological safety.
**Appendix S4:** Consolidated criteria for reporting qualitative studies (COREQ): 32‐item checklist.

## Data Availability

Research data are not shared.
