# Supplementary material for: Exploring How the Psychological Safety of Patients Is Impacted by Restrictive Practices in Inpatient Mental Healthcare: A Qualitative Study
Source: Int J Ment Health Nurs. 2025 Oct 29;34(6):e70148. doi: 10.1111/inm.70148 (PMC12570778; doi:10.1111/inm.70148)
Supplement: Supplementary file 1 — Appendix S1: Topic guide. Appendix S2: Analysis considerations and procedure. Appendix S3: Iterations and development process of the definition of psychological safety. Appendix S4: Consolidated criteria for reporting qualitative studies (COREQ): 32‐item checklist. [file INM-34-0-s001.docx]

**Supplementary Material**

**Appendix S1.** Topic guide

**Appendix S2.** Analysis considerations and procedure

**Appendix S3.** Iterations and development process of the definition of psychological safety.

**Appendix S4.** Consolidated criteria for reporting qualitative studies (COREQ): 32-item checklist

**Appendix S1.** Topic guide

**Exploring the impact of restrictive practice on psychological safety: a qualitative study of the service user experience**

This study aims to explore your experience of restrictive practice and how this impacted your psychological safety. We would also like to know what you think could be done as an alternative to restrictive practice or how it could be made safer. By exploring what you and other people who have experienced or witnessed restrictive practice think, we hope to identify the impacts of restrictive practice on psychological safety to aid improvements to care on inpatient mental health wards. I am going to be recording the interview today, is that ok? Any questions before we get started?

**Topic Guide**

- Before we get started, please tell me a little bit about yourself and your time in inpatient mental health care…
  - Cover: Time spent in mental health wards, number of inpatient stays, type of ward, whether they have direct experience or witnessed restrictive practice.
- The main aspect of this research is the concept of psychological safety. Previous research within the team has shown that feelings of psychological safety in service users was influenced by:

1. Healthcare staff attitudes and behaviours towards them
2. Their relationships with other service users
3. Whether they felt they had any control over their environment and medical decision-making regarding their care
4. Their experiences of physical safety, feeling listened to and believed
5. Access to meaningful occupation on the wards

- In reference to an inpatient stay, what does psychological safety mean to you? Does the above resonate with you?

**Restrictive practice experience**

- Could you tell me about a time where you experienced or witnessed restrictive practice?
  - Who was involved? What happened before and afterwards? How did you feel?
- Do you think your psychological safety was impacted by this incident? Why?
  - Did it change how you felt about staff/ward/care?
- Did you discuss the incident with anyone afterwards?
  - Was there a chance to debrief?
- How do you think the incident could have been managed differently to help you feel safer?

**Alternative approaches**

- Thinking of the time/s you experienced/witnessed restrictive practice, do you think it was necessary in this/these situations? Why?
  - What do you think could’ve been done so this practice was less harmful to your sense of psychological safety?
- If you could speak to staff/policy makers, what suggestions would you give for making restrictive practice more psychologically safe in inpatient mental healthcare?
  - What could be implemented or changed on the ward to reduce the psychological harm of restrictive practice or make it unnecessary?

**Appendix S2.** Analysis considerations and procedure

**Consideration of the Relationship Between Researcher and Participants**

There has been much discussion around the potential power imbalance between interviewer and interviewee in qualitative research (Råheim et al., 2016). While the researcher acknowledges this, steps were taken to reduce the burden on participants. To build rapport with participants in the current study, there was initial contact over email. Here, participants had the opportunity to ask questions, state preferences, and request the topic guide in advance. Before the interview began, the researcher re-introduced themselves stating that they were a PhD student with no prior experience as an inpatient or as staff in mental health services. Participants were reassured that the interviewer wanted to hear their story and were happy for the participant to discuss any experience of restrictive practices that they felt comfortable with. Participants had the opportunity to view and comment on the results of the study once analysis was completed.

The first author of this study was a novice qualitative researcher with no experience of interviewing or working with the target population. As such, the first three interviews were carried out by a more experienced researcher with the first author present on the call. Participants were told from their first contact with the first author that one of her supervisors would be conducting the interview with her present on the call. This was also included in the consent process and reiterated at the beginning of the call. Participants were given the option to withdraw if they did not feel comfortable with this.

**Positioning the research team**

With the complexity of the research area, it is important to consider the research group’s position during the analysis of the data. The lead researcher, a female PhD student does not have experience of working in or being a patient of inpatient mental healthcare. She has received extensive training of qualitative methods during her studies (BSc (Hons), MSc and current PhD study). The research team has extensive experience working in and researching inpatient mental healthcare and restrictive practices.

It was important to consider the wider UK mental healthcare context and the participant’s experience within this. As a result, a critical realist perspective was used throughout the analysis. Critical realism acknowledges the realities of the participant but considers that the experience of an individual does not exist in isolation and are because of relationships and interactions with external structures (i.e., ward environment, staff skills and policy) that have influenced those experiences and realities (Fletcher, 2017).

**Reflexive Thematic Analysis**

**Phase One: Familiarisation with the Data.** The lead researcher led the analysis. Familiarisation started with reviewing each participant’s file, listening to the recording in full whilst reading the transcripts. On the second listen-through, notes on the participants’, and the researcher’s, emotions were made in the author’s reflexivity journal. Transcripts were then printed and then re-read multiple times, making initial notes on contextual information (i.e., type of restrictive practices discussed) on the transcripts and reflections in the reflexivity journal.

**Phase Two: Coding.** The coding process started on paper, naturally taking a more semantic approach (example: realised they’ve been lied to, feeling upset). The qualitative data analysis software nVivo was then used, revisiting the first iteration of codes on paper and developing them. This incorporated more latent coding and reflecting on the initial interpretation. A systematic approach to coding was taken starting with the first interview, making initial codes on paper, moving to nVivo and then going on to the second interview and so on until all transcripts had been coded. It was important to not fit quotes into the codes created on another transcript, but rather naming them individually. Once a point where coding felt complete was reached, the codes were revisited to make sure the code labels accurately reflected the contents. At this point, initial grouping of codes that held the same meaning (i.e., feeling isolated and left alone with feelings) was started.

**Phase Three: Generating Initial Themes.** Clusters were developed through identifying shared meaning across codes. During this process, the researcher reflected on the codes developed, their influence on the coding, and how the codes relate to the research question. An MS Word table was created with the headings: cluster description, codes, quotes and notes. An example cluster description was, “Patients feel punished by restrictive practices and see it as a reflection of staff’s feelings towards them”. Thirteen clusters were created and captured in the table, with quotes to support the cluster. A meeting with the supervisory team was then held to discuss the process and identified clusters. This allowed for a touchpoint on the interpretation of the codes and quotes before moving to developing the themes fully. The clusters and their reflections on their own experiences of certain stories was discussed. The supervisory team also gave feedback on whether the quotes identified represented that cluster and identified areas for further interpretation.

**Phase Four: Developing and Reviewing Themes.** All 13 clusters were revisited, splitting them apart and re-grouping through further interpretation. Similarly, the quotes to support the clusters were revisited, ensuring that the original meaning was maintained in the groupings. This process led to four candidate themes (CT), three of which contained potential subthemes (ST): CT1) physical risk and reactive care (ST 1: Decisions fuelled by physical risk, ST2: Reactive over proactive care), CT2) Chaos is not conducive to safety, CT3) Something around power dynamics (ST1: Power means staff behaviour is interpreted as punishment, ST2: Power in patients looks like rebelling) and CT4) Something about relationships in a closed environment (ST1: The closed nature of the ward, ST2: Relationships are a double-edged sword). The initial interpretations in the first iteration of categories were revisited to ensure that the developed candidate themes were representative of the data. Another meeting with the supervisory team, to discuss the ‘candidate themes’, was had. Feedback focused on whether each candidate theme was reasonably developed, based on the categories discussed in the previous meeting, and that the identified quotes were represented well. Potential names and areas for further interpretation were also discussed.

**Phase Five: Refining, Defining and Naming Themes.** The contents of the themes were developed based on revisiting the developed MS Word tables. The codes and quotes of each category were revisited and cross-referenced to further develop the interpretation for each theme. The original transcripts were reviewed to ensure that the developed themes represented the original words of participants. Reflections during this process focused on the questions, “What story does this theme tell?” and “How does this fit into the overall story about the data?”.

**Phase Six: Writing Up.** The writing-up process began by drafting a synopsis for each theme and discussing how they are related to the research aims. The writing process was iterative in that the original categories were cross referenced, and the ordering of each concept was revisited several times. Once a full draft was written, it was shared with the supervisory team for feedback. This finalised the names of the themes. Several edits and drafts, with input from the research team, have been done to develop an analytic narrative and coherent story, accompanied by the participants quotes, to address the research question.

**Appendix S3.** Iterations and development process of the definition of psychological safety.

**Iteration 1:** Participants in the interview study described what psychological safety means to them. Below is what they said in the context of being an inpatient in mental healthcare.

- Being psychologically safe is being protected from lasting psychological damage by the environment. It’s not just about feeling physically safe on the ward but being protected from things that are going to have lasting effects in the future (iatrogenic harm).
- Psychological safety is having your needs understood – having my diagnosis accepted and adjustments made to make me feel safe, having people not make adjustments repeatedly makes me feel unsafe.
- Providing psychologically safe care is about time, understanding, reflection and empathy. It’s for someone to make you feel like what you’re experiencing is valid.
- Psychologically safe care could be an umbrella term for care that is recovery focused, trauma informed and individualised to service users’ needs.
- Trust that you’re going to be cared for when you’re distressed.
- Psychological safety is feeling safe in yourself, having trust in your own decisions. Providing psychologically safe care would involve encouraging independence and choice.
- Being psychologically safe is being informed about what is happening externally and being able to process and react.

**Feedback and answers to questions from lived experience advisory group**

- Being psychologically safe is being protected from lasting psychological damage by the environment. It’s not just about feeling physically safe on the ward but being protected from things that are going to have lasting effects in the future (iatrogenic harm).
- Psychological safety is having your needs understood – having my diagnosis accepted and adjustments made to make me feel safe, having people not make adjustments repeatedly makes me feel unsafe.
- Providing psychologically safe care is about time, understanding, reflection and empathy. It’s for someone to make you feel like what you’re experiencing is valid.
- Psychologically safe care could be an umbrella term for care that is recovery focused, trauma informed and individualised to service users’ needs.
- ~~Trust that you’re going to be cared for when you’re distressed.~~
- Psychological safety is feeling safe in yourself, having trust in your own decisions. Providing psychologically safe care would involve encouraging independence and choice.
- ~~Being psychologically safe is being informed about what is happening externally and being able to process and react.~~

1. Do you agree with any of the statements above?

- Like the idea of being validated, think it’s the basis of feeling safe
- Reassurance that your experience and needs are valid are needed to feel safe
- Consideration of physical safety, two separate things but needed to feel psychologically safe

1. Is there any that you disagree with, or think could be changed in any way?

- Last bullet point is more of a suggestion for providing psychological safety rather than the feeling itself
- Trust one is separate – it’s an element of being safe but is also a definition of itself. Also, shouldn’t just be cared for when distressed

1. Is there anything that you think could be added?

- Validation that being an inpatient is ok, validation that feeling how you feel as an inpatient is ok.
- Reassurance that you will be cared for as you are, including disabilities, individual needs etc.

1. Do you have any suggestions for a clear, shortened definition for psychological safety?

- When you’re presenting this to staff, it should be split into feeling psychologically safe is… and providing psychologically safe care means…

**Iteration 2:** A consolidated definition based on the interview study, incorporating feedback from the lived experience advisory group. The suggestion for providing psychologically safe care comes from the interview study and literature on psychological safety:

Psychological safety is feeling validated in your experience of the world and the belief you will be treated fairly based on your individual needs. Being psychologically safe provides protection from lasting psychological harm from your environment. It’s not just about being physically safe on the ward but being protected from things that are going to have lasting effects in the future.

Providing psychologically safe care includes:

- Being recovery focused, trauma informed and providing individualised care for service users’ needs.
- Fully explaining decisions and allowing space for patients to ask questions.
- Encouraging the independence and choice of patients.
- Providing a safe space for patients to express feelings and frustrations.
- Providing a physically safe environment.
- Providing access to meaningful occupation on the wards.

**Feedback from lived experience advisory group**

- Good that the underlying message of providing psychologically safe care is around relationships – important to be supported.
  - Element of mutual trust in self and others to enable confidence in patients but also staff to make decisions
- Changes:

1. Remove ‘on the ward’ from the definition – psychological safety refers to everyone/people and the bullet points cover psychological safety in that specific setting
2. Replace ‘things’ with events
3. Change ‘are going to’ to ‘may’
4. Be consistent in terminology – ‘patients’
5. Add a sentence – This definition recognises the need for individualised care. After speaking with a group of experts by experience we are using the term patients to refer to anyone who is receiving care.

**Iteration 3:**

Psychological safety is feeling validated in your experience of the world and the belief you will be treated fairly based on your individual needs. Being psychologically safe provides protection from lasting psychological harm from your environment. It’s not just about being physically safe but being protected from events that may have lasting effects in the future.

Providing psychologically safe care in inpatient mental health includes:

1. Being recovery focused, trauma informed and providing individualised care for patients’ needs.
2. Fully explaining decisions and allowing space for patients to ask questions.
3. Encouraging the independence and choice of patients.
4. Providing a safe space for patients to express feelings and frustrations.
5. Providing a physically safe environment.
6. Providing access to meaningful occupation on the wards.

After speaking with a group of experts by experience we are using the term patients to refer to anyone who is receiving care. We recognise that this is an individual preference and that some people use the terms ‘service user’ or ‘client’.

**Appendix S4.** Consolidated criteria for reporting qualitative studies (COREQ): 32-item checklist

Developed from:

Tong A, Sainsbury P, Craig J. Consolidated criteria for reporting qualitative research (COREQ): a 32-item checklist for interviews and focus groups. *International Journal for Quality in Health Care*. 2007. Volume 19, Number 6: pp. 349 – 357

| **No. Item** | **Guide questions/description** | **Reported on Page #** |
| --- | --- | --- |
| **Domain 1: Research team and reﬂexivity** |  |  |
| *Personal Characteristics* |  |  |
| 1. Interviewer/facilitator | Which author/s conducted the interview or focus group? | 5 |
| 2. Credentials | What were the researcher’s credentials? E.g. PhD, MD | Title page |
| 3. Occupation | What was their occupation at the time of the study? | Supplementary material S2. |
| 4. Gender | Was the researcher male or female? | 6 and supplementary materials S2. |
| 5. Experience and training | What experience or training did the researcher have? | Inferred based on credentials (title page) and mentioned supplementary material S2 |
| *Relationship with participants* |  |  |
| 6. Relationship established | Was a relationship established prior to study commencement? | Supplementary material S2 |
| 7. Participant knowledge of the interviewer | What did the participants know about the researcher? e.g. personal goals, reasons for doing the research | Supplementary material S2 |
| 8. Interviewer characteristics | What characteristics were reported about the inter viewer/facilitator? e.g. Bias, assumptions, reasons and interests in the research topic | Supplementary material S2 |

| **Domain 2: study design** |  |  |
| --- | --- | --- |
| *Theoretical framework* |  |  |
| 9. Methodological orientation and Theory | What methodological orientation was stated to underpin the study? e.g. grounded theory, discourse analysis, ethnography, phenomenology, content analysis | 4 |
| *Participant selection* |  |  |
| 10. Sampling | How were participants selected? e.g. purposive, convenience, consecutive, snowball | 5 |
| 11. Method of approach | How were participants approached? e.g. face-to-face, telephone, mail, email | 5 |
| 12. Sample size | How many participants were in the study? | 7 |
| 13. Non-participation | How many people refused to participate or dropped out? Reasons? | 7 |
| *Setting* |  |  |
| 14. Setting of data collection | Where was the data collected? e.g. home, clinic, workplace | 6 |
| 15. Presence of non-participants | Was anyone else present besides the participants and researchers? | N/A |
| 16. Description of sample | What are the important characteristics of the sample? e.g. demographic data, date | 7 and table 1 |
| *Data collection* |  |  |
| 17. Interview guide | Were questions, prompts, guides provided by the authors? Was it pilot tested? | 6, supplementary material S1/2 |
| 18. Repeat interviews | Were repeat interviews carried out? If yes, how many? | N/A |
| 19. Audio/visual recording | Did the research use audio or visual recording to collect the data? | 6 |
| 20. Field notes | Were ﬁeld notes made during and/or after the inter view or focus group? | Supplementary material S2 |
| 21. Duration | What was the duration of the inter views or focus group? | 7 |
| 22. Data saturation | Was data saturation discussed? | N/A |
| 23. Transcripts returned | Were transcripts returned to participants for comment and/or correction? | N/A |
| **Domain 3: analysis and ﬁndings** |  |  |
| *Data analysis* |  |  |
| 24. Number of data coders | How many data coders coded the data? | Supplementary material S2 |
| 25. Description of the coding tree | Did authors provide a description of the coding tree? | N/A |
| 26. Derivation of themes | Were themes identiﬁed in advance or derived from the data? | Analysis method reported page 5 and supplementary material S2 |
| 27. Software | What software, if applicable, was used to manage the data? | Supplementary material S2 |
| 28. Participant checking | Did participants provide feedback on the ﬁndings? | Supplementary material S2 |
| *Reporting* |  |  |
| 29. Quotations presented | Were participant quotations presented to illustrate the themes/ﬁndings? Was each quotation identiﬁed? e.g. participant number | 8-15 |
| 30. Data and ﬁndings consistent | Was there consistency between the data presented and the ﬁndings? | Yes |
| 31. Clarity of major themes | Were major themes clearly presented in the ﬁndings? | Yes |
| 32. Clarity of minor themes | Is there a description of diverse cases or discussion of minor themes? | N/A |

N/A = Not applicable
